# Retraction: Rebamipide Promotes the Regeneration of Aspirin-Induced Small-Intestine Mucosal Injury through Accumulation of β-Catenin

**DOI:** 10.1371/journal.pone.0297646

**Published:** 2024-02-02

**Authors:** The PLOS ONE Editors

After this article [1] was published, concerns were raised about results presented in Figs 3–5. Specifically:

- In Fig 3A, the upper section of the Con-Reb 5-day panel appears similar to the lower section of the Con-Sal 0-day panel.
- There appear to be multiple instances of vertical discontinuities between lanes in all western blot panels in Figs 3D, 4D and 5E, and the vertical discontinuities do not appear to be in consistent positions across all panels within each figure.
- In Fig 4C, the lower right section of the COX-1 SII-Reb 10-day panel appears similar to the lower left section of the COX-1 SII-Sal 5-day panel.

Contrary to the declaration in the Data Availability statement, the original raw data files supporting the article’s results were not provided with the article. The authors did not respond to queries about the experiments in Figs 3A, 3D, 4D and 5E. In the absence of the underlying data for the panels of concern, the *PLOS ONE* Editors consider that these issues cannot be resolved.

The nature of the issues observed in these images raises concerns regarding the reliability and integrity of the reported results and conclusions. Therefore, the *PLOS ONE* Editors retract this article.

All authors either did not respond directly or could not be reached.

## References

[1] Lai Y, Zhong W, Yu T, Xia Z-S, Li J-Y, Ouyang H, et al. (2015) Rebamipide Promotes the Regeneration of Aspirin-Induced Small-Intestine Mucosal Injury through Accumulation of β-Catenin. PLoS ONE 10(7): e0132031. doi: 10.1371/journal.pone.0132031 26135128 PMC4489841

